# Environmental Hotspots for Azole Resistance Selection of *Aspergillus fumigatus*, the Netherlands

**DOI:** 10.3201/eid2507.181625

**Published:** 2019-07

**Authors:** Sijmen E. Schoustra, Alfons J.M. Debets, Antonius J.M.M. Rijs, Jianhua Zhang, Eveline Snelders, Peter C. Leendertse, Willem J.G. Melchers, Anton G. Rietveld, Bas J. Zwaan, Paul E. Verweij

**Affiliations:** Wageningen University and Research, Wageningen, the Netherlands (S.E. Schoustra, A.J.M. Debets, J. Zhang, E. Snelders, B.J. Zwaan);; Radboud University Medical Center; Nijmegen, the Netherlands (A.J.M.M. Rijs, E. Snelders, W.J.G. Melchers, P.E. Verweij);; Center for Agriculture and Environment, Culemborg, the Netherlands (P.C. Leendertse);; Center of Expertise in Mycology Radboudumc/CWZ, Nijmegen (W.J.G. Melchers, P.E. Verweij);; National Institute for Public Health and the Environment, Bilthoven, the Netherlands (A.G. Rietveld)

**Keywords:** azole resistance, Aspergillus fumigatus, fungi, fungal infections, environmental hotspots, exposure, fungicide, aspergillosis, selection, antimicrobial resistance, the Netherlands

## Abstract

Azole resistance is a major concern for treatment of infections with *Aspergillus fumigatus*. Environmental resistance selection is a main route for *Aspergillus* spp. to acquire azole resistance. We investigated the presence of environmental hotspots for resistance selection in the Netherlands on the basis of the ability of *A. fumigatus* to grow and reproduce in the presence of azole fungicide residues. We identified 3 hotspots: flower bulb waste, green waste material, and wood chippings. We recovered azole-resistant *A. fumigatus* from these sites; all fungi contained *cyp51A* tandem repeat–mediated resistance mechanisms identical to those found in clinical isolates. Tebuconazole, epoxiconazole, and prothioconazole were the most frequently found fungicide residues. Stockpiles of plant waste contained the highest levels of azole-resistant *A. fumigatus*, and active aerobic composting reduced *Aspergillus* colony counts. Preventing plant waste stockpiling or creating unfavorable conditions for *A. fumigatus* to grow in stockpiles might reduce environmental resistance burden.

*Aspergillus fumigatus* is a saprophytic mold whose natural habitat is decaying plant material (*1*). This fungus can tolerate high temperatures (>50°C) that can occur in heaps of decaying plant material. The fungus releases large amounts of aerial asexual spores. Humans might inhale hundreds of *A. fumigatus* spores daily, but aspergillosis generally does not develop in healthy persons because the spores are eliminated by the innate immune response (*1*). However, in immunosuppressed patients, the fungus can cause a range of clinical syndromes ranging from allergic conditions to acute and chronic invasive pulmonary aspergillosis. Invasive aspergillosis is a life-threatening infection that has a mortality rate of up to 60% (*2*).

Triazoles are the main class of drugs for treatment of aspergillus diseases. Clinically licensed anti-*Aspergillus* compounds include itraconazole, voriconazole, posaconazole, and isavuconazole. The triazoles are recommended for prevention of invasive aspergillosis in high-risk patients (posaconazole), for treatment of chronic pulmonary aspergillosis (itraconazole), and for treatment of invasive aspergillosis (voriconazole and isavuconazole). Alternative treatment options are limited to liposomal amphotericin B or echinocandins for specific aspergillus diseases and host groups.

In 1998, triazole-resistant *A. fumigatus* isolates were found in the Netherlands; since then, resistance has been reported from many countries worldwide (*3*,*4*). It is generally accepted that resistance can develop through patient treatment (patient route) and through exposure of *A. fumigatus* to azole fungicides in the environment (environmental route) (*2*–*9*). Environmental resistance mutations commonly are composed of a tandem repeat (TR) in the promoter region of the *cyp51A* gene in combination with single or multiple point mutations in the CYP51A protein (TR_34_/L98H; TR_53_; TR_46_/Y121F/T289A) (*3*,*6*,*10*,*11*). Although *A. fumigatus* is not a phytopathogen and thus not a target for fungicide application, many azole fungicides show in vitro activity against *A. fumigatus* (*12*,*13*). These fungicides include various classes of azoles, such as triazoles (e.g., epoxiconazole), imidazoles (e.g., prochloraz and imazalil), and triazolinthiole (e.g., prothioconazole).

We previously demonstrated that 5 azole fungicides from the triazole class were highly active against wild-type *A. fumigatus* but showed no activity against resistant isolates with TR_34_/L98H (*12*). These 5 azole fungicides showed similarities with the molecular structure of medical triazoles and cross-resistance. This finding complements other studies, which showed that various azole fungicides can induce cross-resistance to medical triazoles because all of these compounds affect the CYP51A enzyme that is central in the ergosterol biosynthesis pathway (*13*,*14*). Patients with triazole-resistant aspergillus disease have a high probability of treatment failure (*15*). The day 42 mortality rate for patients with voriconazole-resistant invasive aspergillosis was found to be 21% higher than for patients with voriconazole-susceptible infections; mortality rates were up to 90% (*16*).

Because genetic diversity, population size, and selection pressure are critical for adaptation of fungi to new environments, we hypothesized that sites that support the growth, reproduction, and genetic variation of *A. fumigatus* and contain residues of azole fungicides would facilitate the emergence, amplification, and spread of triazole-resistance mutations. Locations that meet these 2 criteria were considered hotspots for azole resistance of *A. fumigatus.* In this study, we investigated hotspots as possible sources for selection and reservoirs of triazole-resistant *A. fumigatus* in the environment and aimed to identify and characterize environmental sites that facilitate triazole-resistance selection in *A. fumigatus*.

## Methods

An expert panel suggested potential sites meeting hotspot criteria. The panel included experts from organizations representing husbandry, agriculture, composting, sustainable farming, drinking-water research, fungicide authorization, public health, and medicine. The areas of expertise included pesticides, biocides, *Aspergillus* taxonomy, evolutionary biology, fungal genetics, livestock farming, human and animal *Aspergillus* disease, and plant bulb diseases. The experts formulated that, to support growth of *A. fumigatus*, dead plant biomass should be present because this is the preferred substrate. *A. fumigatus* can grow at a wide range of temperatures (12°C–65°C; 35°C is considered the optimal growth temperature); it prefers high relative humidity (85%–100% is optimal); it is not affected by low pH (3.7–7.6); and it has potential for dispersal from the hotspot of environmental spread of conidia. To enable genetic diversity to arise, we anticipated that sufficient time for reproduction to take place would be a factor that might also affect the ability to select for resistance. In addition, the presence of azole-fungicide residues was considered to be a critical factor to impose selection pressure for azole resistance. The azole residues should furthermore exhibit activity against *A. fumigatus*. Several potential sites fulfilling the criteria were listed and ranked according to the expert estimation of resistance selection risk.

### Sampling of Potential Hotspots

Sites that were identified as potential hotspots were sampled 2 or 3 times (Table). If possible, contrasting sites were selected and compared (i.e., those with known application of azole fungicides and similar sites where no azole fungicides were used).

**Table:** Characteristics of sampling sites in environmental hotspots tested for azole resistance in *Aspergillus fumigatus*, the Netherlands

Sampling site	Type*	Total, CFU/g	Azole-resistant, CFU/g	Resistance mutations (no. colonies)	Azole fungicide (mg/L)†
Wheat grain					
Dry	R	<20	<20	NT	Epoxiconazole; tebuconazole
Moist	R	<20	<20	NT	Epoxiconazole; tebuconazole
Organic	O	<20	<20	NT	NT
Poultry manure	R	<20	<20	NT	NT
	O	<20	<20	NT	NT
Cattle manure	R	<20	<20	NT	Tebuconazole (0.3); epoxiconazole (0.048); prochloraz (0.017); prochlorazdesimidazole-amino (0.34)
	O	<20	<20	NT	Tebuconazole (0.08); epoxiconazole (0.051); proprioconazole (0.038); prothioconazole-destio (0.0205); difenoconazole
Horse manure/straw	R	<20	<20	NT	Tebuconazole (0.082); prochlorazdesimidazole-amino (0.048); epoxiconazole (0.021); cyproconazole (0.012)
	O	<20	<20	NT	Tebuconazole (0.015)
Maize silage					
With Retengo‡	R	<20	<20	NT	Epoxiconazole (0.012)
Without Retengo‡	R	<20	<20	NT	NT
Fruit waste	R	<20	<20	NT	NT
	O	<20	<20	NT	Tebuconazole
Exotic fruit waste	R	<20	<20	NT	Imazalil (0.22)
	O	<20	<20	NT	NT
Household waste, privately owned	R	6.4 × 10^4^	1.5 × 10^3^	TR_34_/L98H (10); TR_34_/L98H/S302N (1)	NT
Household waste, privately owned, duplicate	R	2.7 × 10^5^	1.8 × 10^3^	NT	NT
Household waste, privately owned	O + R	100	<100	NT	Prothioconazole; epoxiconazole
Household waste, privately owned	O	<20	<20	NT	NT
Household waste, fresh	R	2.3 × 10^3^	200	NT	Iprodion (0.024)
Household waste, after 1 wk	R	<100	<100	NT	Propiconazole (0.013); difenoconazole
Household waste, after 3 wk	R	200	<100	NT	Propiconazole (0.021); tebuconazole (0.012); difenoconazole
Flower bulb waste					
Location 1	R	2 × 10^4^	4.3 × 10^3^	TR_34_/L98H (6); TR_46_/Y121F/T289A (7)	Prothioconazole (6.4); prothiconazoledestio (0.15); prochloraz (0.11); prochloraz-desimidazole-amino (0.085)
	O + R	1.2 × 10^4^	2.5 × 10^3^	TR_34_/L98H (7); TR_34_/L98H/F495I (1); TR_46_/Y121F/T289A (2); TR_92_/Y121F/M172I/T289A/G448S (1)	Prothioconazole (1.9); prochloraz (0.09); prochloraz-desimidazole-amino (0.086); prothiconazole-destio (0.07)
Location 2	O + R	2.9 × 10^3^	180	TR_34_/L98H (4); TR_34_/L98H/S297T/F495I (1); TR_46_/Y121F/T289A/I36 4V (1)	Prothioconazole (0.43); prochloraz (0.044); prothiconazole-destio (0.026); epoxiconazole; azaconazole
	O + R	2.7 × 10^4^	3.9 × 10^3^	TR_34_/L98H (5); TR_46_/Y121F/T289A (3)	Prochloraz (0.18); 2,4,6-trichloorphenol (0.17); prothioconazole (0.033); prothiconazole-destio (0.01); tebuconazole (0.022) prochloraz (0.011)
Location 2, 1 y later	O + R	9.4 × 10^5^	2.3 × 10^5^	TR_34_/L98H (5); TR_46_/Y121F/T289A (6); TR_92_/Y121F/M172I/T289A/G448S (1)	Prothioconazole (1.3); prothiconazoledestio (0.033); difenoconazole; epoxiconazole; penconazole; propriconazole; azaconazole; tebuconazole
	O + R	8 × 10^3^	10^3^	NT	Prothioconazole (1.1); prochloraz (0.043); prothiconazole-destio (0.026); epoxiconazole; azaconazole
Location 3	O + R	3 × 10^5^	9 × 10^4^	NT	Prothioconazole (0.72); prothiconazoledestio (0.026); tebuconazole
Flower bulb fermentation	O + R	20	<20	NT	Prothioconazole (1.9); prothiconazoledestio (0.028); tebuconazole (0.021); epoxiconazole
Green waste, location A, 2015					
0–1 wk old	R	9.4 × 10^4^	8 × 10^3^	TR_34_/L98H (12)	Tebuconazole
5–6 wk old	R	1.9 × 10^6^	8.4 × 10^4^	TR_34_/L98H (2)	Tebuconazole (0.004)
7 wk old	R	2.7 × 10^5^	3.6 × 10^3^	TR_34_/L98H (10); TR_34_/L98H/L343H (1); TR_34_/L98H/E356V (1)	Tebuconazole (0.001)
Green waste, location A, 2016					
5–6 wk old	R	3 × 10^5^	9 × 10^4^	NT	Tebuconazole; prothioconazole
7 wk old	R	2.2 × 10^3^	60	NT	Tebuconazole; prothioconazole
Green waste, location B, 2016					
5–6 wk old	R	7 × 10^4^	2.8 × 10^3^	NT	Tebuconazole; prothioconazole
7 wk old	R	2.2 × 10^3^	60	NT	Tebuconazole
Wood chippings waste					
Location 1, 2015	R	6.2 × 10^4^	3.6 × 10^3^	TR_34_/L98H (18)	Azaconazole (0.53); propioconazole (0.036); tebuconazole (0.013)
	R	10^4^	1.3 × 10^3^	NT	Azaconazole (0.53); propioconazole (0.036); tebuconazole (0.013)
Location 1, 2016	R	3 × 10^5^	6 × 10^3^	NT	Propioconazole (0.022); azaconazole; tebuconazole
Location 2, 2016	R	60	<20	NT	Azaconazole (0.53); propioconazole (0.026); tebuconazole (0.02)

*O, organic cultivation; NT, not tested; O + R, cultivation in transition from regular to organic; R, regular cultivation. †If no fungicide concentration is shown, the concentration could not be quantified (i.e., <0.001 mg/L). ‡Retengo is a fungicide that contains 50 g/L of epoxiconazole.

To recover *A*. *fumigatus* isolates from the outdoor environment, we dissolved 2 g of sample in 8 mL of 0.2 mol/L NaCl, 1% Tween 20, as described (*5*). From this suspension, we plated 100 μL on Sabouraud dextrose agar, as well as on agar plates supplemented with 4 mg/L of itraconazole and incubated at 37°C. We compared the number of colonies on itraconazole-containing and itraconazole-free agar. If colonies were present, we collected 20 colonies from each plate.

We used molecular methods for strain identification and determination of the resistance mechanism as described (*17*–*20*). We determined the full coding sequence of the *cyp51A* gene by PCR amplification and sequencing (*14*). We used the *cyp51A* sequence (GenBank accession no. AF338659) for comparison in detecting mutations.

We sent samples to Eurofins Laboratorium Zeeuws-Vlaanderen B.V. (https://www.eurofinsdiscoveryservices.com) for detection and characterization of fungicide residues and metabolites. The following fungicides were analyzed, which covered commonly and less commonly used azole compounds: azaconazole, bromuconazole, cyproconazole, difenoconazole, epoxiconazole, flusilazole, flutriafole, metconazole, penconazole, propiconazole, prothiconazole, tebuconazole, thiabendazole, cyazofamid, fenamidone, iprodione, triazoxide, imazalil, and prochloraz. In addition, metabolites of prochloraz (2,4,6-trichlorophenol and prochloraz-desimidazole-amino) and prothioconazole (prothioconazole-desthio) were analyzed.

## Results

We selected potential hotspots on the basis of preset criteria. These potential hotspots included waste from flower bulbs, residential household waste, green material, wood chippings, exotic fruit, regional fruit, wheat cereal, horse manure, poultry manure, cattle manure, and maize silage.

### Resistance Levels at Sampling Sites

We sampled 11 sites in duplicate or triplicate (total 41 samples). *A. fumigatus* was detected at levels >10^4^ CFU/g in waste from flower bulbs, green material, and wood chippings. One sample of household waste contained high levels of CFUs of *A. fumigatus*, which was not confirmed on sampling at another site. Azole fungicides and their residues were detected in all but 9 samples (Table).

#### Wheat Cereals

Wheat cereals are sprayed with azole fungicides in conventional farming. The grain is collected and stored in warehouses. The straw is partly used in animal stables and mixed with manure. Different animal manure was collected. We analyzed organic and conventional grain and straw. *A. fumigatus* was not found in grain (dry or moist) or manure from different locations and animals. Azole fungicides were present in some samples (Table).

#### Maize

Maize has been occasionally sprayed with azole fungicides in conventional farming since 2014 and is stored in a silage after harvesting. In this study, we sampled 2 types of silage: sprayed (conventional) with azole fungicides and unsprayed. No *A. fumigatus* was found in maize silage (with or without azole fungicides) (Table). This finding can be explained by anoxic fermentation that normal maize silage undergoes, which creates unfavorable conditions for *A. fumigatus* to grow in. Therefore, maize silage is not considered a hotspot.

#### Regional Fruit Waste and Exotic Fruit Waste

Regional fruit and exotic fruit are sprayed with azole fungicides in conventional farming and commonly follow separate waste chains. Some of this fruit may begin to rot during transportation or storage; this fruit is then separated from healthy fruit in waste heaps. We analyzed organic and conventional fruit waste (regional and exotic). *A. fumigatus* was not found (with or without azole fungicides) (Table). 

#### Household Waste

Household waste (consisting of vegetable, fruit, and garden waste) might contain azole fungicides. This green waste was collected by using household containers, taken to collection stations, and processed by using hydrolysis steps. We sampled household waste at the central collection station simultaneously at 3 positions in the hydrolysis process: just before the start of the process, during the process (1 week into the process), and at the end of the process (3 weeks). This household waste contained *A. fumigatus* at the start (2.3 × 10^3^ CFU total counts and 200 azole-resistant CFUs), but low *A. fumigatus* counts remained at the end of the hydrolysis process (3 weeks) (Table). Residues of various azole fungicides were detected in these samples.

Private garden owners sometimes use household organic waste (consisting of vegetable, fruit, and garden waste) in compost heaps. These heaps might contain residues of azole fungicides. We sampled 2 of these heaps and, from 1, recovered >1 × 10^4^ *A. fumigatus* CFUs, including 1.5 × 10^3^–1.8 × 10^3^ azole-resistant colonies, but no azole fungicide residues were detected (Table). The presence of azole-resistant *A. fumigatus* in the absence of azole residues might be caused by azole-resistant *A. fumigatus* in the materials that were added to the heap, rather than an indication that this sample type is a source of azole resistance. Furthermore, the second privately owned compost heap showed no azole-resistant *A. fumigatus*.

#### Flower Bulb Waste

Flower bulbs are sprayed with or dipped in azole fungicides in conventional farming. Bulb waste (peals, bulb, and leaf waste) are collected and stored before composting. We sampled different bulb waste heaps from an organic grower and a conventional grower. *A. fumigatus* and azole fungicides were found in the flower bulb waste heaps in high quantities; high counts of azole-resistant *A. fumigatus* ranged from 180 to 2.3 × 10^5^ CFU/g (Table). The heap from the organic flower bulb grower also contained high levels of azole fungicides. This grower indicated that he had just started the conversion of his farm to organic flower bulb production and therefore the bulb waste still contained fungicides (from applications in the previous season), which showed that azole fungicides have a high chemical stability. We conducted a second sampling and analysis for the heaps 1 year later and the initial findings were confirmed, in terms of presence of azoles and of *A. fumigatus*.

#### Green Material Waste

Organic waste originating from landscaping (including grass, leaves, shrubs and trees) is used for compost, which is professionally produced in large composting plants at several locations in the Netherlands. Stockpiling of the various waste materials precedes active composting. These stockpiling materials contained residues of azole fungicides, and *A. fumigatus* was found in high quantities; counts of azole-resistant *A. fumigatus* ranged from 60 to 8.4 × 10^4^ CFU/g (Table). Repeated sampling after 5–6 weeks and 7 weeks indicated persistence of high levels of *A. fumigatus*. After grinding, woody material is blended with leaves and grass and monitored for temperature during composting. To ensure even heating and oxygenation, the material is turned at regular intervals. This procedure is known as the pathogen-reduction phase. During the consecutive composting stages, *A. fumigatus* CFUs were reduced to approximately or below detectable limits, and no azole-resistant *A. fumigatus* was found in mature compost.

#### Wood Chippings

Waste of processed wood is collected and stored at some of the professional composting plants. This woody material is a mixture of several kinds of wood (e.g., railway sleepers, wooden boxes, and wooden fences). The processed wood contained traces of azole fungicides, and *A. fumigatus* was found in the wood waste in high quantities: counts of azole-resistant *A. fumigatus* were <6 × 10^3^ CFU/g (Table). We conducted a second sampling and analysis in this stored wood 1 year later at the same location and at another location, and results of this sampling confirmed the initial findings.

### Azole Fungicide Measurements

We analyzed 41 samples for azole fungicides, of which 32 (78%) contained azole residues (Table). The number of compounds detected in individual samples ranged from 1 to 8 (median 2.5) fungicides/sample. The most azole fungicides were found in bulb waste (range 3–7 different compounds/sample). In the 32 positive samples, a fungicide was detected 97 times, including 11 different compounds and 3 metabolites. Tebuconazole was detected most frequently (23 samples), followed by epoxiconazole (11), prothioconazole (10), azaconazole (8), and propiconazole (7) (Table). For 33 of 97 positive results, a fungicide could be detected but not quantified (represented as <0.001 mg/kg). For 64 measurements, the fungicide concentration could be quantified and ranged from 0.001 mg/kg to 6.4 mg/kg (mean 0.3 mg/kg; median 0.036 mg/kg). The 3 hotspots contained different fungicides; prothioconazole was found predominantly in flower bulb waste, tebuconazole mostly in green material waste, and azaconazole in wood chippings.

### Genetic Basis of Resistance

The *cyp51A* gene was characterized for 105 azole-resistant *A. fumigatus* isolates from 10 samples; 5 from flower bulb waste at 3 sites; 3 from organic waste; 1 from household green waste; and 1 from wood chippings compost (Table). We analyzed 50 isolates recovered from flower compost, 37 from green material waste, and 18 from wood chippings waste. All isolates contained *cyp51A* TR-mediated resistance mutations; 19 (18%) isolates contained TR_46_ (1 additional repeat of a 46-bp region in the promoter); 2 isolates (2%) contained TR_92_ (2 additional repeats of a 46-bp region in the promoter), and the remaining isolates contained TR_34_ (1 repeat of a 34-bp region in the promoter) (80%) (Table). We observed additional polymorphisms in the *cyp51A* gene for 5 isolates that contained TR_34_/L98H and for 1 TR_46_/Y121F/T289A isolate (Table). TR_34_ was recovered from all samples, and TR_46_ and TR_92_ were recovered only from flower bulb waste.

## Discussion

We identified 3 hotspots for azole-resistance selection in *A. fumigatus*: waste originating from flower bulbs, green material, and wood chippings. Repeated sampling confirmed growth of *A. fumigatus* and azole-resistant *A. fumigatus* and residues of azole fungicides. Household waste was not classified as a hotspot because resistance and azole residues were not consistently found.

These hotspots contained the highest proportions of azole-resistant *A. fumigatus* isolates and azole fungicide residues. The azole fungicides that were detected in our environmental samples are known to show in vitro activity against *A. fumigatus*; these fungicides include azaconazole, epoxiconazole, tebuconazole, prothioconazole, difenoconazole, propiconazole, cyproconazole, prochloraz, and imazalil (*12*). Although to date numerous studies have investigated environmental samples for the presence of azole-resistant *A. fumigatus*, the presence of azole fungicides in the sample is generally not measured (*21*–*24*). Studies that measured azole fungicide concentrations included a study on the presence of azole-resistant *A. fumigatus* in sawmills and that found a major association between the number of resistant colonies recovered and the concentration of propiconazole (*25*). Another study measured levels of 4 azole fungicides from samples taken in flower fields in Colombia and linked these levels to the presence of azole-resistant *A. fumigatus* (*26*).

Although recovery of azole-resistant *A. fumigatus* from environmental samples indicated the presence of resistance, it does not directly implicate that the resistance emerged in that sample; it is possible that already resistant types had colonized the material and were able to proliferate. To gain more insight into resistance selection, we believe that it is critical to determine *A. fumigatus* growth and phenotype, as well as azole residues simultaneously. This study showed that *A. fumigatus* does not grow in some of the sample types we tested, and that the risk for resistance selection is considered low in these environments at that stage of composting, even when azole fungicides are present. Further studies are needed to determine how resistant mutants arise in a hotspot (i.e., through de novo acquisition or by selection of already existing resistance mutations).

We found that azole fungicides were widely present in samples we tested, indicating a broad selection pressure for *A. fumigatus*. In general, low levels of residues were detected, which challenges the concept of high exposure as the main driver for resistance selection or maintenance, as postulated by Gisi (*27*). We previously compared the resistance dynamics in 2 compost heaps with and without azole exposure (*14*) and found that, in the presence of the azole, the dominant phenotype of the *A. fumigatus* populations differed from predominantly wild-type in the absence of azoles to predominantly resistant in the presence of azoles (*14*). Exposure to low concentrations of azoles might pose a greater risk for resistance selection than exposure to high concentrations because at lower concentrations, a larger population of fungal cells will be available to produce progeny (*28*). However, a larger population might purport greater competition for nutrients, underscoring the need to further investigate the dynamics of competition between resistant and wild-type strains in these environments that contain low amounts of azoles. Furthermore, in all samples taken from a hotspot, we recovered wild-type *A. fumigatus*, in addition to resistant phenotypes, indicating that the wild-type population can survive in an environment containing azoles. It is highly likely that the azole fungicide concentrations will vary considerably inside a plant-waste heap and will also vary over time, suggesting a dynamic environment.

Our study indicated that the risk for resistance selection varies depending on the local conditions. Thus, analysis of the entire fungicide application cycle is needed. Aerobic composting is efficient in destroying many harmful microorganisms that are pathogenic to humans or plants. We found that *A. fumigatus* does not survive aerobic composting, possibly because of the high (>70°C) temperatures that are reached. Although *A. fumigatus* is a thermotolerant fungus, asexual spores do generally not survive temperatures >60°C. In contrast, the on-farm stockpiling of plant waste at flower bulb farms before industrial composting treatment occurred was found to be a hotspot.

All azole-resistant *A. fumigatus* isolates harbored CYP51A TR-mediated resistance mutations, which are also the dominant mutations found in patients with azole-resistant invasive aspergillosis in the Netherlands (*16*,*29*). National surveillance of clinical isolates in the Netherlands showed that TR-mediated resistance mutations accounted for >80% of the resistance mutations found in the previous 5 years (*29*). The identical resistance mutations found in the environment and in patients suggests a role for these hotspots in patient infections. However, we cannot exclude that other hotspots exist.

Although our chemical analysis covered a wide range of commonly and less commonly used azole fungicides and their residues, we might have missed relevant azole compounds present in sites that contained azole-resistant *A. fumigatus*. Thus, we were unable to determine which proportion of patients are infected through either 1 of the 3 hotspots that we have identified. Such information will be useful in supporting new policies aimed at reducing the environmental burden of resistance. TR_46_ and TR_92_ mutations were recovered only from flower bulb waste, which might be caused by use of specific (combinations of) azole fungicides or concentration of azole residues. Therefore, future studies should include quantification of aerial dispersal of *A. fumigatus* conidia from hotspots and investigate occupational exposure of agricultural workers to azole-resistant *A. fumigatus*.

Azoles are useful in management of *Aspergillus* diseases and also play a major role in control of fungi during global food production and as biocides in nonfood materials. Therefore, a full ban of the use of this class of antifungal compounds is undesirable, but actions are needed to retain the class for both applications. Our study indicates that composting practices (i.e., stockpiling of plant waste) are key to resistance selection in *A. fumigatus*, rather than mere application of azole fungicides to protect against phytopathogenic fungi. One option would be to create conditions that preclude the growth of *A. fumigatus* and thus to turn the hotspot into a coldspot. The climate in the Netherlands provides favorable conditions for *A. fumigatus* to grow in plant waste material, but favorable conditions for *A. fumigatus* might be prevented (e.g., if the heap was shielded from the outdoor environment and stored under dry conditions). Furthermore, we found no *A. fumigatus* in fruit waste that underwent hydrolysis. These conditions in fruit waste might be unfavorable for *A. fumigatus* because of its anaerobic nature, absence of specific nutrients, or competition with unicellular microorganisms. Further studies are needed to systematically compare different composting techniques in relation to the growth of *A. fumigatus* and to design strategies that prevent resistance selection/maintenance in the environment. In addition, the currently available fungicides should be used prudently and require antifungal stewardship in hospitals and for nonmedical applications, thus preventing unnecessary exposure of fungi, including *A. fumigatus*. Similar interventions have been successful for bacterial resistance in reducing resistance rates (*30*). Furthermore, the possibility of replacing azole fungicides with other fungicide classes that do not cause cross-resistance to medical triazoles should be investigated for different applications.

Although we took great care in selecting which sites were sampled as potential hotspots, our study was limited by the number of sites sampled. Other hotspots that might be present in the environment might contribute to the overall extent of environmental resistance. We believe that our definition of a hotspot and the concept of analyzing the full application cycle of fungicides is broadly applicable and will help to identify additional potential hotspots. Research into azole-resistance selection requires a multidisciplinary approach and can be studied from a One Health perspective. Clarifying in greater detail the factors that drive resistance selection, the dynamics of resistance selection in *A. fumigatus* populations, and the link between hotspot and resistant disease in humans is critical for designing preventive measures.

## References

[1] Latgé J-P. *Aspergillus fumigatus* and aspergillosis. Clin Microbiol Rev. 1999;12:310–50. 10.1128/CMR.12.2.310 10194462 PMC88920

[2] Verweij PE, Chowdhary A, Melchers WJ, Meis JF. Azole resistance in *Aspergillus fumigatus*: can we retain the clinical use of mold-active antifungal azoles? Clin Infect Dis. 2016;62:362–8. 10.1093/cid/civ885 26486705 PMC4706635

[3] Denning DW, Venkateswarlu K, Oakley KL, Anderson MJ, Manning NJ, Stevens DA, et al. Itraconazole resistance in *Aspergillus fumigatus.* Antimicrob Agents Chemother. 1997;41:1364–8. 10.1128/AAC.41.6.1364 9174200 PMC163916

[4] Snelders E, van der Lee HA, Kuijpers J, Rijs AJ, Varga J, Samson RA, et al. Emergence of azole resistance in *Aspergillus fumigatus* and spread of a single resistance mechanism. PLoS Med. 2008;5:e219. 10.1371/journal.pmed.0050219 18998768 PMC2581623

[5] Snelders E, Huis In ’t Veld RA, Rijs AJ, Kema GH, Melchers WJ, Verweij PE. Possible environmental origin of resistance of *Aspergillus fumigatus* to medical triazoles. Appl Environ Microbiol. 2009;75:4053–7. 10.1128/AEM.00231-09 19376899 PMC2698372

[6] Verweij PE, Snelders E, Kema GH, Mellado E, Melchers WJ. Azole resistance in *Aspergillus fumigatus*: a side-effect of environmental fungicide use? Lancet Infect Dis. 2009;9:789–95. 10.1016/S1473-3099(09)70265-8 19926038

[7] Zhang J, Debets AJ, Verweij PE, Melchers WJ, Zwaan BJ, Schoustra SE. Asexual sporulation facilitates adaptation: The emergence of azole resistance in *Aspergillus fumigatus.* Evolution. 2015;69:2573–86. 10.1111/evo.12763 26315993

[8] Camps SM, van der Linden JW, Li Y, Kuijper EJ, van Dissel JT, Verweij PE, et al. Rapid induction of multiple resistance mechanisms in *Aspergillus fumigatus* during azole therapy: a case study and review of the literature. Antimicrob Agents Chemother. 2012;56:10–6. 10.1128/AAC.05088-11 22005994 PMC3256077

[9] Howard SJ, Cerar D, Anderson MJ, Albarrag A, Fisher MC, Pasqualotto AC, et al. Frequency and evolution of Azole resistance in *Aspergillus fumigatus* associated with treatment failure. Emerg Infect Dis. 2009;15:1068–76. 10.3201/eid1507.090043 19624922 PMC2744247

[10] van der Linden JW, Snelders E, Kampinga GA, Rijnders BJ, Mattsson E, Debets-Ossenkopp YJ, et al. Clinical implications of azole resistance in *Aspergillus fumigatus*, The Netherlands, 2007-2009. Emerg Infect Dis. 2011;17:1846–54. 10.3201/eid1710.110226 22000354 PMC3311118

[11] van der Linden JW, Camps SM, Kampinga GA, Arends JP, Debets-Ossenkopp YJ, Haas PJ, et al. Aspergillosis due to voriconazole highly resistant *Aspergillus fumigatus* and recovery of genetically related resistant isolates from domiciles. Clin Infect Dis. 2013;57:513–20. 10.1093/cid/cit320 23667263

[12] Snelders E, Camps SM, Karawajczyk A, Schaftenaar G, Kema GH, van der Lee HA, et al. Triazole fungicides can induce cross-resistance to medical triazoles in *Aspergillus fumigatus.* PLoS One. 2012;7:e31801. 10.1371/journal.pone.0031801 22396740 PMC3291550

[13] Faria-Ramos I, Farinha S, Neves-Maia J, Tavares PR, Miranda IM, Estevinho LM, et al. Development of cross-resistance by *Aspergillus fumigatus* to clinical azoles following exposure to prochloraz, an agricultural azole. BMC Microbiol. 2014;14:155. 10.1186/1471-2180-14-155 24920078 PMC4061453

[14] Zhang J, van den Heuvel J, Debets AJM, Verweij PE, Melchers WJG, Zwaan BJ, et al. Evolution of cross-resistance to medical triazoles in *Aspergillus fumigatus* through selection pressure of environmental fungicides. Proc Biol Sci. 2017;284:20170635. 10.1098/rspb.2017.0635 28931745 PMC5627189

[15] Meis JF, Chowdhary A, Rhodes JL, Fisher MC, Verweij PE. Clinical implications of globally emerging azole resistance in *Aspergillus fumigatus.* Philos Trans R Soc Lond B Biol Sci. 2016;371:20150460. 10.1098/rstb.2015.0460 28080986 PMC5095539

[16] Lestrade PP, Bentvelsen RG, Schauwvlieghe AFAD, Schalekamp S, van der Velden WJFM, Kuiper EJ, et al. Voriconazole resistance and mortality in invasive aspergillosis: a multicenter retrospective cohort study. Clin Infect Dis. 2018; [Epub ahead of print]. 30307492 10.1093/cid/ciy859

[17] Schoustra SE, Debets AJM, Slakhorst M, Hoekstra RF. Mitotic recombination accelerates adaptation in the fungus *Aspergillus nidulans.* PLoS Genet. 2007;3:e68. 10.1371/journal.pgen.0030068 17465683 PMC1857732

[18] Samson RA, Visagie CM, Houbraken J, Hong SB, Hubka V, Klaassen CH, et al. Phylogeny, identification and nomenclature of the genus *Aspergillus.* Stud Mycol. 2014;78:141–73. 10.1016/j.simyco.2014.07.004 25492982 PMC4260807

[19] Debets AJ. Parasexuality in fungi: mechanisms and significance in wild populations. In: Bridge PD, Couteaudier Y, Clarkson JM, editors. Molecular variability of fungal pathogens. Wallingford (UK): CAB International; 1998. p. 41–52.

[20] Schoustra S, Hwang S, Krug J, de Visser JA. Diminishing-returns epistasis among random beneficial mutations in a multicellular fungus. Proc Biol Sci. 2016;283:e20161376. 10.1098/rspb.2016.1376 27559062 PMC5013798

[21] Bader O, Tünnermann J, Dudakova A, Tangwattanachuleeporn M, Weig M, Groß U. Environmental isolates of azole-resistant *Aspergillus fumigatus* in Germany. Antimicrob Agents Chemother. 2015;59:4356–9. 10.1128/AAC.00100-15 25941229 PMC4468681

[22] Lavergne R-A, Morio F, Favennec L, Dominique S, Meis JF, Gargala G, et al. First description of azole-resistant *Aspergillus fumigatus* due to TR_46_/Y121F/T289A mutation in France. Antimicrob Agents Chemother. 2015;59:4331–5. 10.1128/AAC.00127-15 25918139 PMC4468656

[23] Toyotome T, Fujiwara T, Kida H, Matsumoto M, Wada T, Komatsu R. Azole susceptibility in clinical and environmental isolates of *Aspergillus fumigatus* from eastern Hokkaido, Japan. J Infect Chemother. 2016;22:648–50. 10.1016/j.jiac.2016.03.002 27050399

[24] Santoro K, Matić S, Gisi U, Spadaro D, Pugliese M, Gullino ML. Abundance, genetic diversity and sensitivity to demethylation inhibitor fungicides of *Aspergillus fumigatus* isolates from organic substrates with special emphasis on compost. Pest Manag Sci. 2017;73:2481–94. 10.1002/ps.4642 28618166

[25] Jeanvoine A, Rocchi S, Reboux G, Crini N, Crini G, Millon L. Azole-resistant *Aspergillus fumigatus* in sawmills of Eastern France. J Appl Microbiol. 2017;123:172–84. 10.1111/jam.13488 28497646

[26] Alvarez-Moreno C, Lavergne R-A, Hagen F, Morio F, Meis JF, Le Pape P. Azole-resistant Aspergillus fumigatus harboring TR_34_/L98H, TR_46_/Y121F/T289A and TR_53_ mutations related to flower fields in Colombia. Sci Rep. 2017;7:45631. 10.1038/srep45631 28358115 PMC5372364

[27] Gisi U. Assessment of selection and resistance risk for demethylation inhibitor fungicides in *Aspergillus fumigatus* in agriculture and medicine: a critical review. Pest Manag Sci. 2014;70:352–64. 10.1002/ps.3664 24123539

[28] Van de Bosch F, Paveley N, Shaw M, Hobbelen P, Oliver R. The dose debate: does the risk of fungicide resistance increase or decrease with dose? Plant Pathol. 2011;60:597–606. 10.1111/j.1365-3059.2011.02439.x

[29] DeGreef S, Mouton J. NethMap 2017: consumption of antimicrobial agents and antimicrobial resistance among medically important bacteria in the Netherlands. MARAN 2017: monitoring of antimicrobial resistance and antibiotic usage in animals in the Netherlands in 2016 [cited 2019 May 16]. https://www.rivm.nl/publicaties/nethmap-2017-consumption-of-antimicrobial-agents-and-antimicrobial-resistance-among

[30] Speksnijder DC, Graveland H, Eijck IAJM, Schepers RWM, Heederik DJJ, Verheij TJM, et al. Effect of structural animal health planning on antimicrobial use and animal health variables in conventional dairy farming in the Netherlands. J Dairy Sci. 2017;100:4903–13. 10.3168/jds.2016-11924 28390724

